# Management of Twig Canker and Shoot Blight of Almond Caused by *Diaporthe amygdali*: Efficacy of Chemical Fungicides and Biological Control Agents

**DOI:** 10.3390/jof12090700

**Published:** 2026-09-18

**Authors:** Carolina Francia, Josep Armengol, Antonio Ramón, Mónica Berbegal

**Affiliations:** 1 Instituto Nacional de Investigación Agropecuaria, INIA La Estanzuela, Colonia 70000, Uruguay; cfrancia@inia.org.uy; 2 Instituto Agroforestal Mediterráneo, Universitat Politècnica de València, 46022 Valencia, Spain; jarmengo@eaf.upv.es (J.A.); anraal@etseamn.upv.es (A.R.)

**Keywords:** biocontrol, *Prunus dulcis*, nut crops, disease management

## Abstract

This study evaluated the in vitro and *in planta* efficacy of six chemical fungicides (boscalid, captan, copper oxychloride, difenoconazole, pyraclostrobin, and potassium hydrogen carbonate) and two biological control agents (*Bacillus amyloliquefaciens* subsp. *plantarum* D747 and *Trichoderma atroviride* strain SC1) against *Diaporthe amygdali*, the causal agent of twig canker and shoot blight disease of almond. In vitro assays demonstrated that *D. amygdali* is highly sensitive to captan, difenoconazole, and pyraclostrobin. Regarding biological products, dual antagonism assays showed high efficacy for *T. atroviride*. Subsequently, the pre- and post-infection activity of all products was assessed on one-year-old almond plants. Efficacy varied significantly depending on the timing of application. *Trichoderma atroviride* was effective at all application times, achieving mean percent disease control (MPDC) values of 100% when applied preventively. Pre-infection applications of difenoconazole and pyraclostrobin resulted in 100% MPDC. However, their efficacy progressively decreased when applied post-infection. *Bacillus amyloliquefaciens* and captan showed variable results, reaching a maximum of 73% and 87% MPDC, respectively, when applied before inoculation. In contrast, boscalid, copper oxychloride, and potassium hydrogen carbonate exhibited very low MPDC values, regardless of application timing. These findings provide valuable information for improving management strategies against *D. amygdali* in almond orchards.

## 1. Introduction

Traditionally, almond cultivation in Spain has been considered marginal, frequently associated with suboptimal soils, reliant on rainfed conditions, and managed with minimal inputs, resulting in low yields [1]. However, in recent years, the increasing market value of almonds has transformed this crop into a highly profitable crop when grown in suitable soils with proper agricultural practices [2,3,4].

The intensification of almond cultivation has introduced new phytosanitary challenges, including a rising incidence of pathogens that reduce crop productivity [5,6,7]. Among these pathogens, *Diaporthe amygdali* has been identified as a significant threat to almond production in the Mediterranean Basin [8,9,10,11,12].

*Diaporthe amygdali* is the causal agent of twig canker and shoot blight disease of almond trees [8,10,13]. The symptoms of this disease develop in late winter or early spring with rapid drying of shoots, flowers, and leaves. Brown lesions are initially observed around the buds of green shoots and later progress into annual cankers, sometimes accompanied by a gummy exudate, along with twig wilting. In severe attacks, *D. amygdali* can lead to tree defoliation [13].

The management of twig canker and shoot blight in almond orchards has primarily relied on calendar-based phytosanitary treatments, which are adjusted according to the phenological stages of the crop [1,14]. Currently available control options are limited due to the low number of fungicides authorized for this crop. In Spain, although a threshold of 5% affected buds has been proposed as a management guideline, no fungicide treatments have proven curative efficacy under field conditions once infection is established [1,15].

Several studies have evaluated the in vitro and *in planta* efficacy of fungicides against *D. amygdali*, affecting both almond and peach trees [14,16,17,18,19,20,21,22]. In peach orchards, Lalancette and Robison (2002) [14] observed that autumn fungicide treatments reduced canker incidence by 45–63%, while spring applications achieved only 10–28% control. Chlorothalonil and captan were most effective when applied in both seasons, reducing canker incidence by 46–71% [14]. Schnabel and Lalancette (2003) [19] established a classification of fungicide efficacy against *D. amygdali* in peach, indicating that chlorothalonil, captan, thiram, and ferbam were the most effective, in that order. Thomidis et al. (2009) [20] demonstrated that fungicides such as iprodione, carbendazim, thiophanate-methyl, and tebuconazole completely inhibit mycelial growth of *D. amygdali* peach isolates in vitro. ZhaoLin et al. (2013) [22] reported prochloraz to be the most effective fungicide against *D. amygdali* in peach both in vitro and *in planta*, providing 90–95% control of infected twigs in field trials, while carbendazim and difenoconazole achieved 70–80% efficacy. More recently, Feng et al. (2020) [16] investigated the use of biofungicides against *D. amygdali* isolated from peach, demonstrating that D-limonene-loaded nanoemulsions inhibited *D. amygdali* growth by 51.7% with an EC_50_ of 35.74 μg/mL.

Regarding *D. amygdali* management on almonds, Tuset et al. (1997) [21] assessed the efficacy of fungicides including benomyl, imazalil, and methyl-thiophanate, reporting reductions in disease severity of 65%, 35%, and 55%, respectively, when spraying the leaves of almond seedlings. Moreover, Rhouma et al. (2008) [18] evaluated the efficacy of benomyl, carbendazim, methyl-thiophanate, copper, and procymidone. Their results showed that benomyl, carbendazim, and methyl-thiophanate completely inhibited mycelial growth and conidial germination of *D. amygdali* isolated from almond in vitro, and in field trials located in Tunisia, these fungicides reduced infected shoots by more than 70% [18].

While fungicides are known for their benefits in crop production, including increased yields, improved consistency, and enhanced profitability, they also pose significant risks to human health and the environment due to their toxicity and persistence [23]. Furthermore, overuse of fungicides potentially leads to resistance development, threatening long-term efficacy and food security [24,25].

Biological control represents a sustainable alternative that may help to reduce the application rates of fungicides as part of an integrated management strategy. The use of Biological Control Agents (BCAs) against *D. amygdali* has been scarcely explored. Rhouma et al. (2008) [18] showed that *Trichoderma viride* and *T. harzianum* reduced the mycelial growth of *D. amygdali* by around 25% and decreased the incidence of infected buds by over 50% compared to untreated control, when applied to almond trees both in autumn and spring. In addition, a bacterial suspension of *Bacillus licheniformis* W10 and its antifungal protein completely inhibit mycelial growth, conidial germination and germ-tube elongation of *D. amygdali* from peach, and reduce sporulation [26].

*Diaporthe amygdali* presents a broad infection risk period, as α-conidia can germinate and infect almond trees over a wide range of temperatures (between 5 and 35 °C), allowing infections to occur year-round [27]. Furthermore, many almond cultivars have been reported to be susceptible or highly susceptible to the pathogen [28,29], probably because most of them are derived from a few elite cultivars used intensively in breeding programs worldwide [30].

Recently, genomic analyses of *D. amygdali* have revealed candidate virulence genes and other factors involved in pathogenicity. The identification of a broad range of biosynthetic gene clusters for secondary metabolites, including those for known phytotoxins and potential novel compounds, along with a large set of candidate effectors, highlights the metabolic versatility and complexity of the *D. amygdali* secretome [31].

For these reasons, the management of *D. amygdali* on almond relies on cultural practices and fungicide treatments according to a schedule adjusted by phenological stages of the crop [1,14,19]. However, these treatments are limited to preventive applications made before flowering and leaf autumn, based on the assessed risk of the orchard and local weather conditions, due to their lack of curative efficacy [15]. Lalancette and Robison (2002) [14] considered that an effective fungicide program for *D. amygdali* would provide at least 90% disease control, but studies performed with different products on peach and almond so far have reported much lower efficacy [16,18,19,21]. Additionally, several of the fungicides evaluated in previous studies have been banned, further reducing chemical control options, and resistance of *D. amygdali* to thiophanate-methyl, boscalid and fluopyram has been reported [32,33].

Implementing more sustainable management practices, especially those that incorporate biological control, may help to reduce the incidence and severity of diseases affecting almond trees, an issue of great importance for both growers and the almond industry [34]. In the specific case of twig canker and shoot blight disease caused by *D. amygdali*, further research is crucial to enhance our knowledge and to develop more effective control strategies. Therefore, the objectives of this study were: (i) to evaluate the in vitro efficacy of chemical fungicides and biological control agents (BCAs) currently in use against *D. amygdali* isolates obtained from almond; and (ii) to determine their pre- and post-infection effectiveness on almond plants.

## 2. Materials and Methods

### 2.1. Fungal Isolates

Two fungal isolates of *D. amygdali*, PHAL-4 and PHAL-45, obtained from almond trees in Spain showing symptoms of twig canker and shoot blight disease and previously characterized [10] were used in this study. They were stored in 15% glycerol solution at −80 °C in cryovials (1.5 mL) in the fungal collection of the Instituto Agroforestal Mediterráneo—Universitat Politècnica de València (Spain). Isolates were grown on potato dextrose agar (PDA; Biokar-Diagnostics, Zac de Ther, France) for 7 days at 25 °C in the dark before the experiments. Both isolates were used for the in vitro evaluation of fungicides and biological control agents (BCAs), while for the evaluation of pre- and post-infection activity on almond plants, only PHAL-45 was used.

### 2.2. Chemical Fungicides and Biological Control Agents

Six chemical fungicides were evaluated in vitro to determine their efficacy in inhibiting mycelial growth and conidial germination of *D. amygdali.* Additionally, two BCAs were tested to assess their antagonistic activity against this pathogen (Table 1). The active ingredients and BCAs evaluated were selected based on their authorization for use in almond production and/or for the control of the disease in Spain, according to MAPA (2025) [35] at the time the experiment was conducted.

### 2.3. Mycelial Growth and Conidial Germination Assay

For mycelial growth evaluation, appropriate volumes of each fungicide were added to potato dextrose agar (PDA; Condalab, Madrid, Spain) to achieve final concentrations of 0.1, 1, 10 and 100 mg a.i. L^−1^. Mycelial plugs (6 mm in diameter), obtained from the margins of 7-day-old actively growing cultures of *D. amygdali* isolates PHAL-4 and PHAL-45, were transferred to fungicide-amended plates. Each fungicide concentration and isolate had five replicates. Control PDA plates were prepared similarly but with sterile distilled water added instead of the fungicide solution. Fungicide-amended plates were incubated in the dark at 25 °C for 5 days, after which the diameter of each colony was measured twice perpendicularly. Measurements were made at the same time and averaged. The experiment was performed twice.

For conidial germination evaluation, colonies of *D. amygdali* were incubated at 25 𠂰C with a photoperiod of 12 h light and 12 h darkness for 30 days to obtain mature pycnidia. A conidial suspension was obtained for each isolate by flooding the agar surface with 12 mL of sterile distilled water and scraping with a sterile spatula. The resulting spore suspension was filtered through two layers of cheesecloth into an Erlenmeyer flask (250 mL). The filtrate was diluted with sterile water, and conidial concentration was adjusted with a hemocytometer to 10^5^ α conidia mL^−1^. Appropriate volumes of each fungicide were added to PDA to obtain a final concentration of 0.1, 1, 10 and 100 mg of active ingredient per liter (mg a.i. L^−1^). Two 20 µL drops of the conidial suspensions were deposited on one PDA Petri plate. Five replicates per isolate and per concentration of each fungicide were assessed, and the experiment was performed twice. Control PDA plates were prepared similarly, but with sterile distilled water added instead of the fungicide solution. The plates were incubated for 24 h at 25 °C in the dark. After 24 h, the percentage of germinated conidia was determined by examining 100 conidia in each drop using a microscope (×40 magnification). A conidium was considered germinated when the length of the germ tube was longer than the length of the conidium.

Percentage inhibition of mycelial growth and conidial germination for each isolate at each concentration was calculated as a relative percentage to the corresponding control treatments. Percentages of mycelial growth and conidial germination inhibitions were converted to probits and plotted against log_10_ values of the fungicide concentration. Probit regression analysis was used to calculate the effective concentration values that inhibited mycelial growth and conidial germination by 50% (EC_50_ values). The EC_50_ value was calculated for each isolate and fungicide.

### 2.4. Dual Culture Antagonism Assay

Antagonistic activity of *Trichoderma atroviride* SC1 was evaluated using dual cultures. Mycelium plugs of 6 mm diameter were cut from the growing edge of 7-day-old cultures grown on PDA of *T. atroviride* SC1 and *D. amygdali* isolates PHAL-4 and PHAL-45. Plugs of *T. atroviride* SC1 and *D. amygdali* isolates were plated 1 cm from opposite edges of round 90 mm PDA plates (7 cm apart from each other) along the same diameter. For *Bacillus amyloliquefaciens* subsp. *plantarum* D747 dual culture, one drop of 20 µL prepared according to the application rate was placed opposite the *D. amygdali* mycelium plug as explained before. For both BCAs, Petri plates were incubated at 25 °C in the dark for 11 days. *Diaporthe amygdali* isolates PHAL-4 and PHAL-45 were individually grown without the presence of BCAs under the same conditions as experimental controls. Each BCA/*D. amygdali* combination was replicated five times, and the experiment was performed twice.

**Table 1:** Chemical fungicides and biological control agents used in the experiments.

Active Ingredient	Chemical Group	Trade Name	Manufacturer	Formulation ^1^	Application Rate ^2^	Authorized Against *D. amygdali* ^3^	Authorized on Almond ^3^
Boscalid	Pyridine-carboxamides	Cantus	BASF (Barcelona, Spain)	500 g Kg^−l^ WG	0.2 mL L^−1^ (almonds)	−	+
Captan	Phthalimides	Agrocapt flow	ADAMA (Madrid, Spain)	475 g L^−l^ SC	3 mL L^−1^ (stone fruits)	+	−
Copper oxychloride	Inorganic	Ditiver C	Industrias Químicas del Vallés (Mollet del Vallés, Spain)	500 g Kg^−l^ WP	2 g L^−1^ (almonds)	*+*	+
Difenoconazole	Triazoles	Score	Syngenta (Madrid, Spain)	250 g L^−l^ EC	0.5 mL L^−1^ (almonds)	−	+
Pyraclostrobin	Methoxy-carbamates	Cabrio	BASF (Barcelona, Spain)	200 g Kg^−l^ WG	0.5 g L^−1^ (almonds)	−	+
Potassium hydrogen carbonate	Diverse	Karbicure	Certis Belchim (Elche, Spain)	850 g Kg^−l^ WP	5 g L^−1^ (almonds)	−	+
**Species**	**Strain**	**Trade Name**	**Manufacturer**	**Colony-Forming** **Units (CFU)**	**Application Rate**	**Authorized Against** ***D. amygdali***	**Authorized** **on Almond**
*Trichoderma atroviride*	SC1	Vintec	Certis Belchim (Elche, Spain)	1 × 10^10^ g^−1^ (WG)	0.2 g L^−1^ (almonds)	*+*	+
*Bacillus amyloliquefaciens* subsp. *plantarum*	D747	Amilo X	Certis Belchim (Elche, Spain)	5 × 10^10^ g^−1^ (WG)	0.2 g L^−1^ (almonds)	−	+

^1^ WP, wettable powder; WG, water-dispersible granule; EC, emulsifiable concentrate; SC, suspension concentrate. ^2^ Application rates in Spain were determined according to De Liñán (2024) [36]. ^3^ Authorized according to MAPA (2025) [35].

After the incubation time, the area occupied by each *D. amygdali* isolate was measured using ImageJ software (version 2.14.0) [37] based on a reference distance common to all images. The percent of mycelium growth inhibition was calculated using the formula:

(1) Percent Growth Inhibition PGI = [(B−A)/B] × 100

[M20] in which A is the area of the pathogen grown in dual culture, and B is the area of the pathogen growing without the BCA (control) [38].

### 2.5. Evaluation of Pre- and Postinfection Activity of Chemical Fungicides and Biological Control Agents on Almond Plants

Pre- and post-infection activity against *D. amygdali* was evaluated using 60 cm tall 1-year-old almond plants of cv. “Avijor”, grown on their own roots in plastic pots (220 cc) containing peat moss. Five wounds were made on the stem of each plant by a sterile awl (3 mm in diameter) before the application of the different products in the case of pre-infection treatments, or before the inoculation in the case of post-infection treatments. The first puncture was done on the main stem 15 cm above the surface of the substrate, and four additional punctures were done up in the stem with a separation of 5 cm each. Pre- and post-infection activity of the products was evaluated at different application times: 7, 3, 1, or 0 days before inoculation, or 1, 2 or 3 days after inoculation. These application timings were selected to assess both preventive and potential curative effects.

Products were prepared according to their application rate and were applied with a hand sprayer (W560, Wanger Spraytech Iberica S.A., Molins de Rei, Spain) until runoff. For fungal inoculation, a uniform layer of fine droplets of a conidial suspension adjusted to 10^5^ conidia mL^−1^ of isolate PHAL-45 was sprayed using a hand sprayer (W560, Wagner Spraytech Iberica S.A., Spain). Different types of control plants were prepared depending on the day the wound was made. Five plants were wounded 7 days before inoculation; five plants were wounded 3 days before inoculation; five were wounded one day before inoculation and, finally, five were wounded on the inoculation day. These control plants were inoculated on the same day (day-0) but were not treated and were used to consider the effect of natural wound healing on infection over time.

Following inoculation, the plants were covered for 24 h with plastic bags to ensure leaf wetness. For the day-0 application (day of inoculation), sprayed plants were allowed to dry for one hour prior to inoculation. There were five replicate plants for each product and application time combination. The experiment was conducted in an environmentally controlled greenhouse at 25 °C, where the plants were randomly distributed, and the experiment was performed twice.

**Figure 1:**
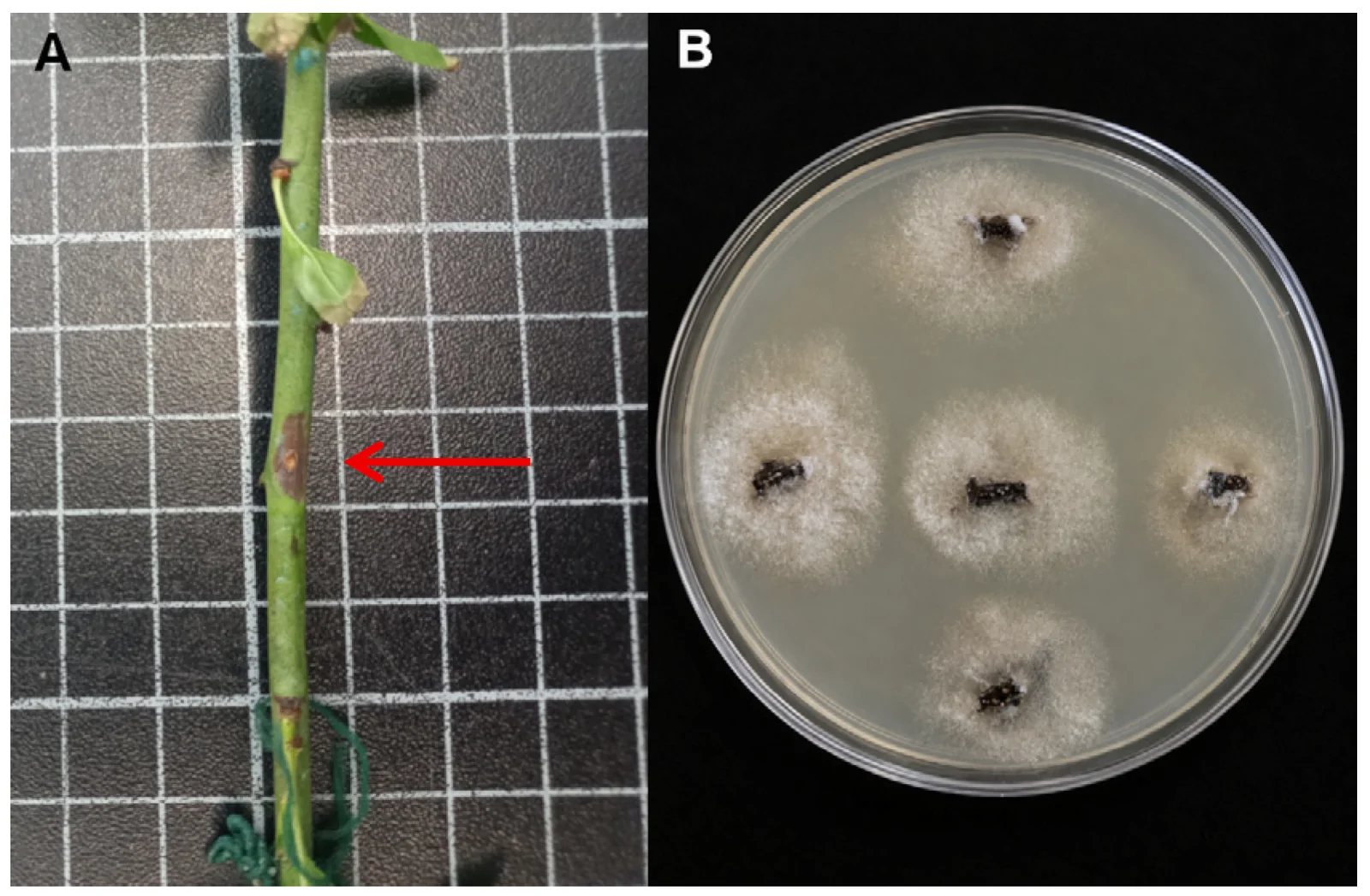
Evaluation of pre- and post-infection activity of chemical fungicides and biological control agents on almond plants. (**A**) Detail of lesion development from the inoculated wound indicated by an arrow; (**B**) Colonies of *Diaporthe amygdaly* growing on PDAS from lesions, after 7 days of incubation at 25 °C.

The number of infected wounds per stem was evaluated 12 days after inoculation. For this purpose, five fragments of stem per plant, each containing one wound, were cut, surface disinfected by dipping them in 70% ethanol for 1 min, washed in sterile water, and then dried in a laminar flow cabinet. The five fragments were placed in one PDA plate amended with 0.5 g L^−1^ of Streptomycin Sulfate (PDAS) to re-isolate the pathogen to determine if the wounds were infected (Figure 1). The percentage of the wounds that were infected was calculated as the number of wounds from which *D. amygdali* was re-isolated divided by the total number of wounds per stem. The data are presented as the mean percent recovery (MPR). The mean percent disease control (MPDC) was calculated based on the MPR of the corresponding control treatments as:

(2) MPDC = 100 × (1 − [MPR_treatment/MPR_control])

### 2.6. Statistical Analysis

Data analyses were performed using RStudio version 2025.09.1+401 [39]. For in vitro experiments, including mycelial growth and conidia germination evaluation (EC_50_ data), the effects of the factors were analyzed using generalized linear mixed models (GLMMs) fitted with a Tweedie distribution and a log link using the *glmmTMB* function from the *glmmTMB* package [40]. Prior to model fitting, no significant differences were detected between experiments by the Kruskal–Wallis test (*p* = 0.089 and *p* = 0.7795, for mycelial growth and conidia germination, respectively). GLMMs were built by considering the products (fungicides and BCAs) and the isolates as fixed factors, and the experiment as a random factor. The dispersion and residuals of the models were tested by using the functions *testDispersion* and *simulateResiduals* of the *DHARMa* package. Once the significance of fixed effects was assessed (Table 2), pairwise comparisons of means were performed using the *emmeans* package [41] with Bonferroni adjustment, and grouping letters were generated using the *cld* function from the *multcomp* package [42].

In the dual culture antagonism assay, the Kruskal–Wallis rank-sum test showed that there were no significant differences between the experiments (Table 3). Thus, data from the two experiments were combined.

For the *in planta* experiment, the effects of product, application time and experiment on MPDC (%) were analyzed using the Kruskal–Wallis rank-sum test by the *kruskal_test* function of the *rstatix* package [43]. Once the significance of effects was assessed, pairwise comparisons of MPDC from different products at different days from inoculation were performed using Dunn’s test with Bonferroni adjustment. Letter groups were generated using the *multcompLetters* function from the *multcompView* package [44].

## 3. Results

### 3.1. Mycelial Growth and Conidial Germination Assay

Significant differences were found among fungicides for both mycelial growth and conidial germination (*p* < 0.01). Mean EC_50_ values for the reduction in *D. amygdali* mycelial growth and conidial germination are shown in Table 4. Captan, difenoconazole, and pyraclostrobin were the most effective fungicides at inhibiting mycelial growth. Their EC_50_ values for isolates PHAL-4 and PHAL-45 were 24.0 and 24.8 mg a.i. L^−1^, 0.22 and 0.14 mg a.i. L^−1^, and <0.1 and 0.1 mg a.i. L^−1^, respectively. Boscalid, copper oxychloride and potassium hydrogen carbonate were not effective in inhibiting mycelial growth of *D. amygdali.*

Regarding conidial germination, captan and pyraclostrobin were the most effective fungicides, with EC_50_ values for isolates PHAL-4 and PHAL-45 of 1.87 and 1.58 mg a.i. L^−1^ and 0.42 and 0.10 mg a.i. L^−1^, respectively. Boscalid, copper oxychloride and difenoconazole were less effective, with EC_50_ values for isolates PHAL-4 and PHAL-45 of 19.79 and 7.68 mg a.i. L^−1^, 11.24 and 8.38 mg a.i. L^−1^, and 5.91 and 5.60 mg a.i. L^−1^, respectively, while potassium hydrogen carbonate was not effective in inhibiting conidial germination of *D. amygdali.* In general, the EC_50_ values obtained between the isolates PHAL-4 and PHAL-45 did not differ substantially, except for spore germination of PHAL-45 with boscalid and copper oxychloride, which showed slightly higher sensitivity than PHAL-4.

**Table 4:** EC_50_ values for the in vitro inhibition of mycelial growth and conidial germination of *Diaporthe amygdali* by the chemical fungicides evaluated.

	Mycelial Growth (EC_50_ ± SE) ^1^		Conidial Germination (EC_50_ ± SE) ^1^	
Fungicide/Isolate	PHAL-4	PHAL-45	PHAL-4	PHAL-45
Boscalid	>100 ± NA ^2^	>100 ± NA	19.79 ± 2.01 e	7.68 ± 0.97 c
Captan	24.0 ± 2.71 c ^3^	24.8 ± 2.77 c	1.87 ± 0.32 b	1.58 ± 0.29 b
Copper oxychloride	>100 ± NA	>100 ± NA	11.24 ± 1.30 d	8.38 ± 1.03 c
Difenoconazole	0.22 ± 0.04 b	0.14 ± 0.02 b	5.91 ± 0.79 c	5.60 ± 0.76 c
Potassium hydrogen carbonate	>100 ± NA	>100 ± NA	>100 ± NA	>100 ± NA
Pyraclostrobin	<0.10 ± 0.003 a	<0.10 ± 0.001 a	0.42 ± 0.1 a	0.10 ± 0.03 a

^1^ Mean EC_50_ values (mg L^−1^) ± standard error. ^2^ NA: not applicable. ^3^ Values followed by different letters in the column are significantly different according to Bonferroni-adjusted pairwise comparisons of means.

### 3.2. Dual Culture Antagonism Assay

**Figure 2:**
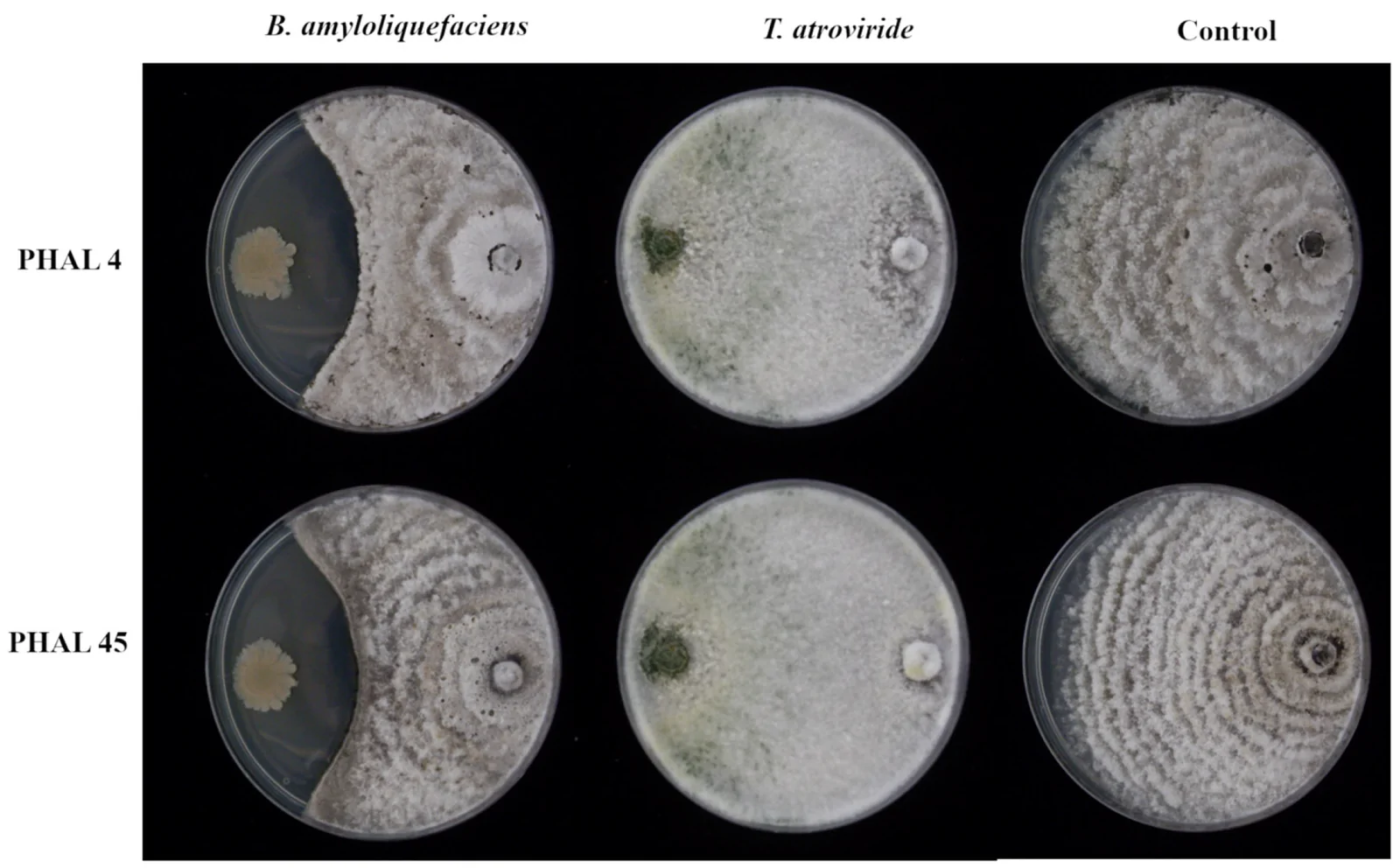
Potato dextrose agar Petri plates showing the antagonistic activity of *Bacillus amyloliquefaciens* subsp. *plantarum* D747 and *Trichoderma atroviride* SC1 against *Diaporthe amygdali* in the dual culture antagonism assay after 11 days of incubation at 25 °C.

Petri plates showing the results of the dual culture antagonism assay with the two BCAs against *D. amygdali* are shown in Figure 2. Significant differences between *B. amyloliquefaciens* subsp. *plantarum* D747 and *T. atroviride* SC1 were observed, with PGI values of 29% and 100%, respectively.

### 3.3. Evaluation of Pre- and Postinfection Activity of Chemical Fungicides and Biological Control Agents

Statistical analysis showed that there were significant differences among the different products and among application times evaluated (Table 5). In addition, there were no significant differences between the experiments.

**Table 5:** Kruskal–Wallis rank-sum test for effects on mean percent disease control (MPDC, %).

	MPDC (%)	
	Df ^1^	Pr (>Chisq)
Application time	6	5.38 × 10^11^
Experiment	1	0.1643
Product	7	<2.2 × 10^−16^

^1^ Degrees of freedom.

**Figure 3:**
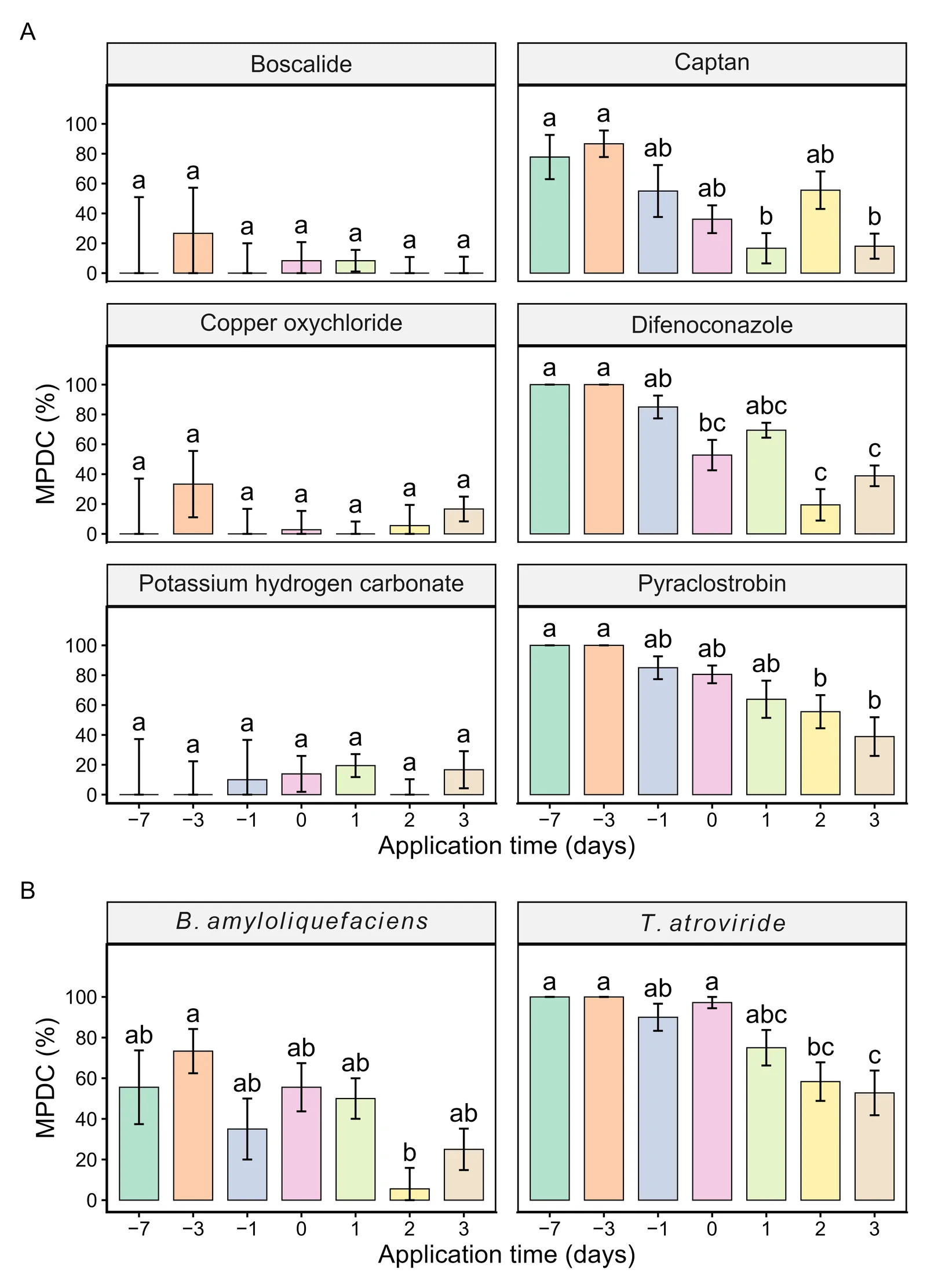
Mean percent disease control (MPDC, %) of eight products applied at different times (pre- and post-infection): (**A**) chemical fungicides and (**B**) biological control agents. Mean percent disease control (MPDC) was calculated as the reduction in mean percent recovery (MPR) relative to the inoculated corresponding control treatment: 100 × (1 − [MPR treatment/MPR control]). Error bars indicate the standard error. Values represented are the mean of ten plants, and different letters within each product show significant differences.

MPR in the non-treated controls increased as the time interval between wounding and inoculation decreased (Supplementary Table S1). Therefore, the analysis was performed relative to the corresponding control. Figure 3 shows the MPDC values of the chemical fungicides and BCAs applied at different times (pre- and post-infection activity). *Trichoderma atroviride* SC1 was effective at all application times evaluated, with MPDC from 90% to 100% when it was applied as preventive, but when it was applied as post-infection, its efficacy was around 60%. Difenoconazole and pyraclostrobin resulted in MPDC values of 100% when they were applied before inoculation. However, when these fungicides were applied after inoculation, their efficacy progressively decreased, reaching MPDC values of 39% for both of them at 3 days after inoculation. *Bacillus amyloliquefaciens* subsp*. plantarum* D747 and captan had variable results, reaching maximum MPDC values of 73% and 87%, respectively, when applied three days before inoculation. Finally, in general, for boscalid, copper oxychloride and potassium hydrogen carbonate MPDC values were very low at most application times (maximum MPDC values of 27%, 33% and 19%, respectively), indicating that these products were the least effective against *D. amygdali* in both pre-infection and post-infection strategies.

## 4. Discussion

The present study evaluated six chemical fungicides and two BCAs, through in vitro and *in planta* experiments, for their potential to control twig canker and shoot blight of almond caused by *D. amygdali*.

The in vitro experiments with chemical fungicides showed that *D. amygdali* was highly sensitive to captan, difenoconazole and pyraclostrobin, which were effective in reducing both mycelial growth and conidial germination. Captan showed intermediate EC_50_ values for mycelial growth, and its performance was better at inhibiting conidial germination. The results obtained with difenoconazole agree with previous in vitro evaluations performed by ZhaoLin et al. (2013) [22] in which difenoconazole showed very low EC_50_ values (0.0692 mg a.i. L^−1^) for the mycelial growth of *D. amygdali* isolates from peach.

Mycelium of *D. amygdali* was not sensitive to boscalid, copper oxychloride and potassium hydrogen carbonate, while conidia germination was slightly affected by boscalid and copper oxychloride. Potassium hydrogen carbonate did not reduce conidia germination even at high concentrations. Rhouma et al. (2008) [18] performed an in vitro evaluation of fungicides using *D. amygdali* isolates from almond, in which copper reduced mycelial growth at high doses but did not completely inhibit it.

When these six chemical fungicides were evaluated for their pre- and post-infection activity using almond plants, our results confirmed that pyraclostrobin and difenoconazole were highly effective against *D. amygdali* when they were applied in a pre-infection strategy, achieving 100% control. However, their efficacy decreased progressively when applied post-infection. Captan showed intermediate performance when it was applied both pre-infection and post-infection. The fungicides boscalid, copper oxychloride and potassium hydrogen carbonate showed, in general, very low effectiveness for the control of *D. amygdali* either in pre-infection or post-infection applications.

Some of the fungicides used in our study against *D. amygdali* had been evaluated previously in field trials. In peach orchards, the results of these studies align with our findings. Lalancette and Robison (2002) [14] showed intermediate effectiveness for captan, as this fungicide is more effective in spring applications compared to autumn applications. ZhaoLin et al. (2013) [22] demonstrated a high efficacy of difenoconazole against shoot blight in this crop. On the other hand, in almond orchards, Rhouma et al. (2008) [18], using a copper-based fungicide, observed a 40% reduction in infected shoots when this fungicide was applied during both the autumn and spring periods.

Regarding BCAs, our results showed that *T. atroviride* SC1 had a high efficacy against *D. amygdali* both in dual culture antagonism assays and *in planta,* being more effective in almond plants when it was used preventively, reaching 100% of control at seven days pre-infection applications. Consistent with our findings, Rhouma et al. (2008) [18] reported a high efficacy of *T. harzianum* and *T. viridae* strains against *D. amygdali* on almond trees in both autumn and spring treatments. *Bacillus amyloliquefaciens* subsp. *plantarum* D747 had intermediate efficacy against *D. amydali* both in vitro and *in planta*.

The results of this study should be interpreted within the context of the experimental conditions, as all inoculations were performed through artificial wounds on one-year-old potted almond plants. Consequently, the efficacy of the product showed performance against wound-mediated infections rather than natural infections. In almond orchards, *D. amygdali* infection and the efficacy of the products may be influenced by inoculum availability and weather conditions [45]. Therefore, further field trials are required to determine whether the efficacies observed here are maintained under a natural infection scenario. In addition, the *in planta* assays were conducted using a single *D. amygdali* isolate, which does not represent variability in fungicide sensitivity and virulence occurring among isolates. Thus, future studies should also include multiple isolates.

Interestingly, fungicides and BCAs showing high efficacy against *D. amygdali* in our study had been previously evaluated to control other important almond diseases [46,47,48]. Holland et al. (2021) [46] evaluated difenoconazole in a mix with azoxystrobin or cyprodinil to protect pruning wounds against almond trunk pathogens such as *Botryosphaeria dothidea*, *Neofusicoccum parvum*, *Cytospora sorbicola*, *Ceratocystis destructans* and *Eutypa lata*, obtaining 66% or 29% efficacy of 66% in a mix with azoxystrobin or cyprodinil, respectively. In addition, Adaskaveg et al. (2022) [49] reported difenoconazole as an excellent and consistent fungicide for the control of brown rot (*Monilinia* spp.) and anthracnose (*Colletotrichum* spp.) in almond. Olmo et al. (2017) [47] demonstrated that pyraclostrobin and boscalid reduced the canker lesions compared with those resulting from the untreated almond pruning wounds against *Botryosphaeriaceae* spp. Adaskaveg et al. (2022) [49] reported that the mix of pyraclostrobin and boscalid was highly effective in controlling brown rot and anthracnose. Moreover, the efficacy of pyraclostrobin and boscalid in a mix was assessed in almond field trials against *M. laxa,* showing an efficacy of 82.4% and 85.2% in two subsequent years [48].

Regarding BCAs, Holland et al. (2021) [46] found that *T. atroviride* SC1 had an efficacy of 90% in protecting almond pruning wounds against trunk pathogens. Adaskaveg et al. (2022) [49] indicated that *B. amyloliquefaciens* had limited or erratic efficacy in controlling brown rot and anthracnose. More recently, Travadon et al. (2023a) [50] evaluated BCAs including *T. atroviride* SC1 using detached almond stems in the laboratory against the canker pathogens *Cytospora plurivora*, *Eutypa lata*, *Neofusicoccum parvum*, and *Neoscytalidium dimidiatum*. The results of the detached-almond-stem experiment indicated that *T. atroviride* SC1 significantly reduced infections by all four pathogens. The abilities of *T. atroviride* were further evaluated by these authors in field trials to evaluate the protection of almond pruning wounds against *E. lata* and *N. parvum*. These results showed that *T. atroviride* protected almond pruning wounds as efficiently as thiophanate-methyl, a well-known, recommended fungicide for treatment of almond pruning wounds [50]. Additionally, Travadon et al. (2023b) [51] assessed commercial BCAs including *B. amyloliquefaciens* MBI 600 and *T. atroviride* SC1 for their ability to prevent almond pruning wound infections *by E. lata* and *N. parvum* under field conditions. The results showed that *B. amyloliquefaciens* treatment was not significantly different from nontreated control plants. In contrast, *T. atroviride* SC1 provided the best protection against *E. lata* and *N. parvum* [51].

The present work provides phenotypic efficacy data for currently available commercial fungicides and well-established commercial biocontrol strains. However, mechanistic aspects, such as the production of volatile or soluble antifungal metabolites, the colonization capacity of biocontrol strains in almond stem tissues, and the induction of host defense responses, were not investigated. Future research should address these mechanisms and explore how chemical fungicides interfere with the physiological processes involved in *D. amygdali* infection.

## 5. Conclusions

Our findings provide important insights on the efficacy of captan, difenoconazole, and pyraclostrobin, both in vitro and *in planta*, supporting their potential use for the management of *D. amygdali* in almond. We also demonstrate that the use of BCAs is an alternative tool for the management of this disease, with *T. atroviride* being highly promising. As only one isolate was used in this study, it should be considered that the results may not be representative of the entire pathogen population. These results, together with previous evidence of efficacy against other relevant almond fungal diseases, highlight that different effective products can be combined to design more sustainable integrated pest management strategies, alternating chemical fungicides and BCAs in almond orchards.

## Figures and Tables

**Table 2:** Likelihood ratio (Chi-square) tests for fixed effects in the generalized linear mixed models (GLMMs) on mycelial growth and conidial germination of *Diaporthe amygdali*.

EC50	Mycelial Growth		Conidial Germination	
	Df ^a^	Pr (>Chisq)	Df ^a^	Pr (>Chisq)
Chemical fungicide	2	<2.2 × 10^−16^	4	<2.2 × 10^−16^
Isolate	1	0.84	1	4.8 × 10^−9^
Chemical fungicide × isolate interaction	4	4.6 × 10^−5^	4	2.4 × 10^−4^

^a^ Degrees of freedom.

**Table 3:** Kruskal–Wallis rank-sum test for fixed effects on mycelial growth of *Diaporthe amygdali*.

	EC_50_	
	Df ^1^	Pr (>Chisq)
Percent growth inhibition		
BCA	1	7.33 × 10^−9^
Experiment	1	0.7504
Isolate	1	0.2033
BCA		PGI ^2^
*B. amyloliquefaciens*		29 b ^3^
*T. atroviride*		100 a

^1^ Degrees of freedom. ^2^ Mean values of percent growth inhibition (%). ^3^ Values followed by different letters in the column are significantly different according to Bonferroni-adjusted pairwise comparisons of means.

## Data Availability

The raw data supporting the conclusions of this article will be made available by the authors on request.

## Supplementary Materials

The following supporting information can be downloaded at: https://www.mdpi.com/article/10.3390/jof12090700/s1, Table S1: Mean percent recovery (MPR) of *Diaporthe amygdali* in the non-treated controls according to the days comprised between wounding and inoculation.
