# Supplementary material for: Management of Twig Canker and Shoot Blight of Almond Caused by Diaporthe amygdali: Efficacy of Chemical Fungicides and Biological Control Agents
Source: J Fungi (Basel). 2026 Sep 18;12(9):700. doi: 10.3390/jof12090700 (PMC13608810; doi:10.3390/jof12090700)
Supplement: Supplementary file 1 [file jof-12-00700-s001.zip › jof-4533435-supplementary.pdf]

Supplementary

Supplementary Table S1. Mean percent recovery (MPR) of *Diaporthe amygdali* in the non-treated controls according to the days comprised between wounding and inoculation

Days before inoculation	MPR (%)
7	18
3	30
1	40
0	72
